# Network Pharmacology Reveals the Therapeutic Potential of BBB-Permeable Compounds from *Lonicera caerulea* for Alzheimer’s Disease and Lipid Metabolism Disorders

**DOI:** 10.3390/ijms27104556

**Published:** 2026-05-19

**Authors:** Jiayi He, Jihong Li, Junwei Huo, Yijun Pang, Kaiqi Sun, Haoyu Zhu, Yuhan He, Zhixuan Ren, Xin Cheng, Shuang Ao, Yahui Peng

**Affiliations:** 1 Department of Biochemistry and Molecular Biology, School of Basic Medical Sciences, Harbin Medical University, Harbin 150086, China; m05250169@163.com (J.H.); lijh@hrbmu.edu.cn (J.L.); 18065159847@163.com (Y.P.); 13670688217@163.com (K.S.); 2023151051@hrbmu.edu.cn (H.Z.); 17868492618@163.com (Y.H.); rzx13327064460@outlook.com (Z.R.); 15884468410@163.com (X.C.); 2022152117@hrbmu.edu.cn (S.A.); 2 Translational Medicine Center of Northern China, Harbin 150086, China; 3 Key Laboratory of Biology and Genetic Improvement of Horticultural Crops (Northeast Region), Ministry of Agriculture and Rural Affairs, College of Horticulture and Landscape Architecture, Northeast Agricultural University, Harbin 150030, China; huojunwei@neau.edu.cn; 4 National-Local Joint Engineering Research Center for Development and Utilization of Small Fruits in Cold Regions, National Development and Reform Commission, Harbin 150030, China

**Keywords:** *Lonicera caerulea*, Alzheimer’s disease, network pharmacology, molecular docking, lipid metabolism disorders, Mendelian randomization, molecular dynamics simulation

## Abstract

Although risk factors for Alzheimer’s disease (AD) involve obesity and elevated low-density lipoprotein (LDL) cholesterol levels, and *Lonicera caerulea* has been reported to improve lipid metabolism disorders (LMDs), it remains unknown whether *Lonicera caerulea* can simultaneously modulate the progression of both AD and LMDs. In this study, an integrative strategy combining network pharmacology, Mendelian randomization (MR), molecular docking, and molecular dynamics simulations was employed to explore potential targets, pathways, and causal relationships. Network pharmacology and molecular docking results revealed that several blood–brain barrier (BBB)-permeable active components of *Lonicera caerulea*, including Naringenin and Palmatine, may be associated with targets involved in the lipid and atherosclerosis pathway, such as HSP90AA1, SRC and TNF. These associations indicate a potential link between the modulation of lipid metabolism and AD-related processes, although further validation is required. Molecular dynamics simulations were conducted to support the stability of key docking complexes. Given that elevated LDL is a central feature of LMDs and a key indicator of cholesterol imbalance, MR analysis was conducted to assess its causal relationship with AD. The results provided genetic evidence supporting a causal role of elevated LDL in AD risk, reinforcing the epidemiological link between lipid metabolism and neurodegeneration. These findings imply that BBB-permeable constituents of *Lonicera caerulea* may exert multi-target effects relevant to AD and LMDs. Enrichment analysis further indicates a possible involvement of pathways associated with lipid and atherosclerosis, supporting its potential as a dietary strategy for at-risk populations.

## 1. Introduction

Alzheimer’s disease (AD), which accounts for approximately 60–80% of dementia cases overall, is among the major contributors to death worldwide [1,2]. Increasing evidence suggests that beyond the abnormal accumulating amyloid-β plaques and tau tangles, which lead to the death of nerve cells, degeneration of neuron, and damage to brain tissue [3], metabolic disturbances are also highly implicated in AD, revealing a characteristic of multiple mechanism and difficulty in treatment for AD progression [4].

Currently recognized drugs targeting the cholinergic hypothesis for Alzheimer’s disease, such as memantine and donepezil, only selectively ameliorating language, memory, orientation, behavior, and visuospatial abilities, fail to halt or delay AD’s progression [5,6]. While anti-β-amyloid antibody, which effectively target the most toxic Aβ, quickly clear plaques and inhibit their formation [7], such as lecanemab, is excluded from use in AD patients presenting with a history of cerebral hemorrhage or other risk factors for intracranial hemorrhage [8]. Therefore, new intervention strategies with wide applicability and minimal adverse reactions are urgently needed for AD intervention.

It is reported that lipid metabolism disorders (LMDs), involved in obesity, high LDL cholesterol and diabetes, are modifiable risk factors for Alzheimer’s disease [9]. Atherosclerosis, one of the most common results of lipid metabolism disorders [10], has been revealed to increase the risk and severity of Alzheimer’s disease to some extent [11]. However, whether ameliorating LMDs can delay AD progression through some pathways related to atherosclerosis has not been fully elucidated. In addition, identifying more genetic overlaps between LMDs and AD may lead to a clearer relationship and enable more diversified early intervention strategies.

Given the limitations of drug therapy, combining medication with non-pharmacological interventions has become the primary approach for treating Alzheimer’s disease. Notably, dietary intervention stands out as a typical non-pharmacological treatment due to its safety and accessibility [12]. *Lonicera caerulea* is a cold-adapted species native to China, Japan, Russia, and Northern Europe, where its berries have been traditionally consumed as food and medicinal resources [13,14]. It has been shown to inhibit lipid peroxidation and lipid accumulation through suppressing adipogenesis and promoting the beiging of adipocytes [15,16]. Moreover, relevant meta-analyses have indicated that *Lonicera caerulea* can improve vascular function and lower blood pressure [17]. Consistent with its heat-clearing and fire-purging effects documented in traditional Chinese medicine [18], epidemiological evidence suggests that many components of *Lonicera caerulea*, such as (-)-epicatechin, Naringenin, Palmatine and anthocyanin, possess antioxidant, anti-inflammatory, and neuroprotective activities [19,20], giving them the potential to intervene in the progression of Alzheimer’s disease [21,22]. However, it remains unclear whether specific components of *Lonicera caerulea* are associated with certain physiological pathways to ameliorate Alzheimer’s disease and lipid metabolism disorders. It should be noted that the blood–brain barrier (BBB) selectively controls the passage of substances from the bloodstream into the brain, preventing many molecules with potential for treating central nervous system disorders from exerting their effects [23]. Therefore, identifying BBB-permeable compounds from *Lonicera caerulea* is a crucial step in evaluating their actual intervention potential for AD.

When it comes to studying the relationship between multi-component drugs and complex diseases, a more systematic and comprehensive approach is needed. To systematically investigate the relationship between multiple components of *Lonicera caerulea* and AD-LMD, this study combined network pharmacology with Mendelian randomization. Network pharmacology integrates multi-omics data and network analysis techniques to construct multidimensional “Component-Target-Disease (CTD)” interaction networks, thereby systematically elucidating the potential pharmacological mechanisms of drugs [24,25]. Mendelian randomization (MR) can overcome the limitations of hybrid bias and reverse causality in traditional observational studies, inferring causality between various indicators and AD more efficiently and reliably [26]. Molecular docking, based on the induced-fit theory, is a computational simulation method that predicts the optimal binding pose and binding affinity between small-molecule ligands and biological macromolecular receptors at their active sites [27]. Molecular dynamics simulations (MDs) enable all non-hydrogen atoms to evolve under Newtonian mechanics, capturing the dynamic nature of protein–ligand interactions, thereby overcoming limitations of static docking and validating binding stability over time [28]. By combining these approaches, it becomes possible to break through the limitations of a single target and simultaneously target multiple proteins in the disease signaling module, thus contributing to a more comprehensive grasp of the potential interaction mechanisms between AD and LMDs.

In this study, network pharmacology, Mendelian randomization, molecular docking, and molecular dynamics simulation methods were employed to identify potential targets, signaling pathways, and molecular mechanisms by which BBB-permeable components of *Lonicera caerulea* may act on both AD and LMDs. The findings provide a theoretical basis for the rationale of *Lonicera caerulea* in the early prevention of AD and lipid metabolism disorders, offering new insights into clinical management of the diseases.

## 2. Results

### 2.1. Screening Lonicera caerulea Active Components and Obtaining Core Targets

Through LC–MS/MS and literature review, 156 active ingredients of *Lonicera caerulea* were finally obtained and presented in Table S1. The total ion chromatogram is shown in Figure S1.

**Figure 1:**
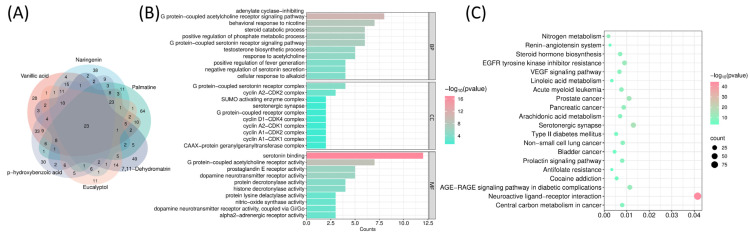
Exploration of the potential functions of components in *Lonicera caerulea* that can pass through BBB. (**A**) Targets Venn of the six effective components in *Lonicera caerulea*. (**B**) Functional annotation and categorization of core targets using Gene Ontology (BPs, CCs, and MFs). (**C**) KEGG pathway enrichment analysis revealed pathways strongly correlated with *Lonicera caerulea*.

As shown in Table 1, based on the oral bio-availability (OB) ≥ 30%, blood–brain barrier (BBB) score ≥ −0.3 and the results from RO5, six effective drug-like components were finally identified, including Eucalyptol (MOL005970), p-hydroxybenzoic acid (MOL000103), Vanillic acid (MOL000114), Naringenin (MOL004328), Palmatine (MOL000785) and 7,11-Dehydromatrin (MOL006583). After deduplication screening, a total of 454 targets were found in the *Lonicera caerulea* active components (Figure 1A).

### 2.2. Prediction of the Main Functions of Lonicera caerulea

Gene Ontology (GO) enrichment analysis (Figure 1B) revealed that the majority of these targets were enriched in biological processes (BPs), including the adenylate cyclase-inhibiting G protein-coupled acetylcholine receptor signaling pathway, steroid catabolic processes, the G protein-coupled serotonin receptor signaling pathway, cellular response to alkaloids, negative regulation of serotonin secretion, and response to acetylcholine. Moreover, the cellular components (CCs) analysis highlighted the G protein-coupled serotonin receptor complex, multiple cyclin complexes, SUMO-activating enzyme complex, G protein-coupled receptor complex, and serotonergic synapse. Additionally, in the molecular functions (MFs) category, *Lonicera caerulea* was found to primarily work by influencing G protein-coupled acetylcholine receptor activity, dopamine neurotransmitter receptor activity, protein decrotonylase activity, histone decrotonylase activity, protein lysine delactylase activity, α2-adrenergic receptor activity, and dopamine neurotransmitter receptor activity via Gi/Go coupling. BP, CC, and MF terms associated with *Lonicera caerulea*’s BBB-permeable components were prominently enriched in GPCR signaling and serotonin-related pathways, indicating that the predicted targets are linked to neuronal signaling and metabolic processes.

Kyoto Encyclopedia of Genes and Genomes (KEGG) pathway enrichment analysis is crucial for further validating these biological functions and inferring the potential pathways influenced by *Lonicera caerulea*’s BBB-permeable components. A total of 169 significant signaling pathways were successfully identified in our study. Subsequently, the top 20 pathways with the highest enrichment scores were selected and visualized in a bubble plot (Figure 1C). Based on primary biological functions and medical relevance, they were systematically categorized into three distinct groups, including neurological psychiatric pathways, metabolic disease-related pathways and cancer-related pathways. First of all, similar to GO enrichment analysis, we identified several pathways associated with neurological and psychiatric disorders in KEGG results. These included the serotonergic synapse, cocaine addiction, and neuroactive ligand-receptor interaction pathways. Especially, as a representative signaling pathway for Alzheimer’s disease, the neuroactive ligand-receptor interaction pathway exhibited the most pronounced enrichment and differed substantially from other pathways [29]. The second group is connected with metabolic disease-related pathways, including type 2 diabetes and its complications (AGE-RAGE pathway), fatty acid metabolism (linoleic acid/arachidonic acid), and the renin-angiotensin system, which regulate glucose and lipid metabolic homeostasis via insulin resistance, oxidative stress, and inflammatory responses. Particularly, linoleic and arachidonic acids (omega-6 fatty acids) were implicated in regulating blood pressure, inflammatory responses, cholesterol balance, and brain function [30,31]. The third group involved cancer-related pathways, including acute myeloid leukemia, prostate cancer, pancreatic cancer, and non-small cell lung cancer.

These results revealed that *Lonicera caerulea* may be related to neuronal signaling dysfunction, metabolic dysregulation, and dysregulated cell cycle control.

### 2.3. Analysis of the Main Targets of Blood–Brain Barrier-Permeable Components from Lonicera caerulea

**Figure 2:**
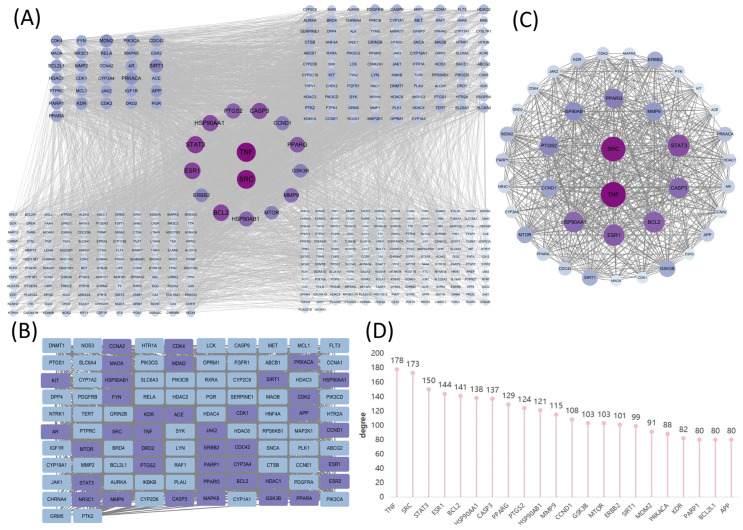
Analysis of the main targets of components in *Lonicera caerulea* that can pass through the blood–brain barrier. (**A**) Selection of core targets in PPI networks, color and size of the targets reflected the degree value. (**B**) Key target screening process by cytoNCA plugin. Targets above the median for degree (>54.5), betweenness centrality (>0.0060), and proximity (>0.4949) are light purple; the rest are blue. (**C**) The 39 hub targets, color and size of the targets reflected the degree value. (**D**) The bar chart of PPI degree values showed top seven targets were TNF, SRC, STAT3, ESR1, BCL2, HSP90AA1 and CASP3.

To visually assess the impact of *Lonicera caerulea*, we constructed a protein–protein interaction (PPI) network consisting of 445 nodes (nine targets have no direct connection with others) and 6308 edges, with an average degree value of 28.35 (Figure 2A). We chose 102 targets whose degree value was greater than twice the median (degree value ≥ 20) to form Figure 2B. And then, targets with degree value, betweenness centrality, and closeness all greater than their respective medians (betweenness centrality > 0.0060, closeness > 0.4949, degree value > 54.5) were selected and colored in light purple. By extracting the purple part separately for analysis, we finally obtained a new PPI network consisting of 39 nodes and 542 edges (Figure 2C). According to the degree values in the PPI network, we further screened seven key targets with a degree > 130 for subsequent analysis, namely TNF, SRC, STAT3, ESR1, BCL2, HSP90AA1 and CASP3 (Figure 2D). These targets exhibited higher connectivity compared to other nodes, intimating that they may occupy central positions in the interaction network and could be functionally relevant in the context of AD and LMDs.

### 2.4. Exploration into the Intrinsic Link Connecting Alzheimer’s Disease to Lipid Metabolism Disorders

**Figure 3:**
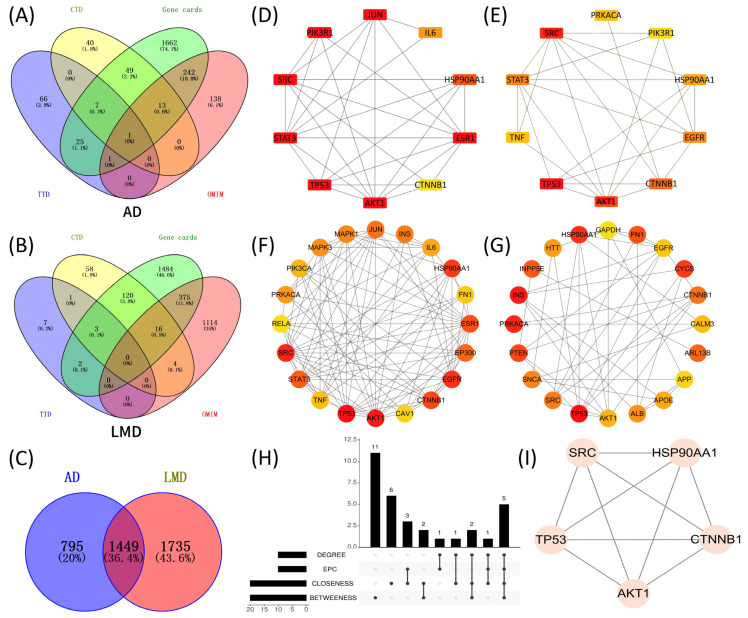
Comorbidity analysis of AD and LMDs. (**A**–**C**) AD and LMD targets were searched from GeneCards, OMIM, CTD, and TTD. Their intersection yielded 1449 common targets. (**D**–**G**) Core targets were separately identified in Cytoscape using Degree, EPC, closeness, and betweeness. (**H**,**I**) By intersecting the targets obtained from the aforementioned four screening criteria in Cyto-Hubba plugin, we obtained the five hub targets that we were most interested in, including HSP90AA1, SRC, TP53, AKT1 and CTNNB1.

Using GeneCards, OMIM, CTD, and TTD databases, 2244 potential targets for Alzheimer’s disease and 3184 for lipid metabolism disorder were identified, with 1449 overlapping genes revealed by a Venn diagram (Figure 3A–C). We got a PPI network of co-expressed targets from STRING database, and then analyzed it through Cyto-Hubba plugin in Cytoscape. By taking the intersection of the top 10 core targets ranked by degree and EPC (Edge Percolated Component), along with the top 20 core targets of closeness and betweenness, five hub targets were identified: SRC, HSP90AA1, TP53, AKT1, and CTNNB1 (Figure 3D–I). Among them, HSP90AA1 and SRC are also the hub targets in the PPI networks of *Lonicera caerulea*. Many core targets were shared, associated with numerous peripheral targets in the PPI network and formed a collective network, indicating a close link between AD and LMDs. Therefore, the study aimed to explore common pathways potentially influenced by *Lonicera caerulea* targeting these diseases.

### 2.5. Genetic Evidence for Causal Relationship Between LDL and AD Risk

**Figure 4:**
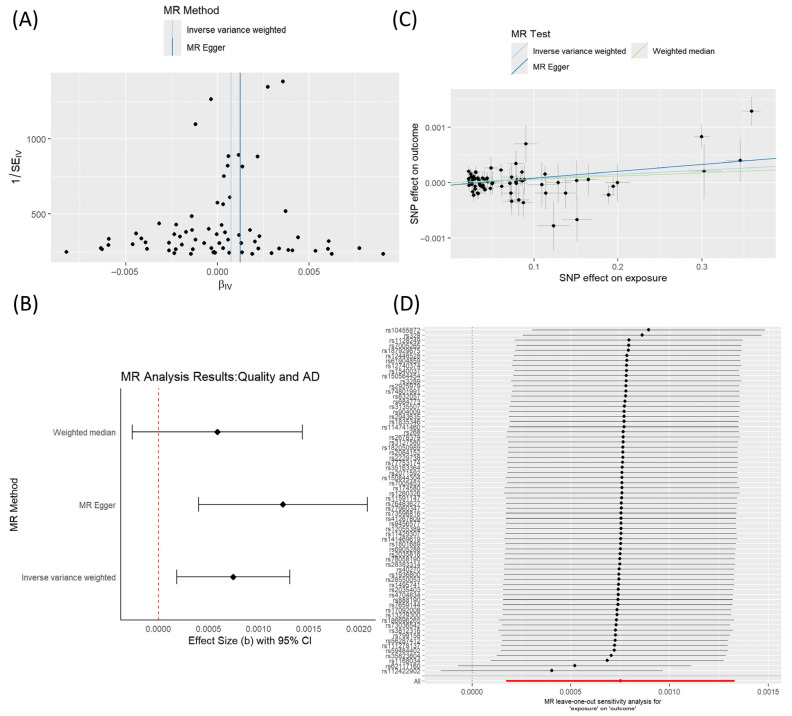
Mendelian randomization analysis of the causal relationship between LDL and AD risk. (**A**) Forest plot of the causal effects of individual SNPs on AD risk. The overall effect estimate from the IVW method is represented by the diamond. (**B**) The red dotted line represents the line of no effect. Funnel plot for assessing the potential pleiotropy. The symmetrical distribution of the SNPs suggests no significant directional pleiotropy. (**C**) Scatter plot of the SNP effects on LDL (*x*-axis) against the SNP effects on AD (*y*-axis). Each point usually represents an individual SNP (single nucleotide polymorphism). The slopes of the lines represent the causal effect estimates from different MR methods. (**D**) Leave-one-out sensitivity analysis. The plot shows the stability of the IVW causal estimate when each SNP is omitted sequentially. The result remained consistent, indicating that the overall finding was not driven by any single influential SNP. The red line represents the combined effect value (point estimate) of all SNPs, used as a reference for sensitivity analysis.

Abnormal levels of LDL are the core marker and direct manifestation of lipid metabolism disorders [32]. We further conducted a Mendelian randomization (MR) analysis to evaluate whether genetically predicted LDL levels are associated with AD risk, thereby providing complementary evidence for a potential link between lipid metabolism and AD. The results are presented in Figure 4.

In this study, we explored the association between genetically predicted LDL levels and AD using MR analysis. A total of 71 SNPs (single nucleotide polymorphisms) associated with LDL were selected as instrumental variables. The funnel plot showed an overall symmetric distribution of the SNP-specific estimates, suggesting no obvious directional asymmetry (Figure 4A). As summarized in Table 2, the inverse variance weighted (IVW) analysis showed a statistically significant positive association between genetically predicted LDL and AD (β = 0.0007458163, SE = 0.0002876124, *p* = 0.009510689). A similar direction of effect was observed in the MR-Egger analysis (β = 0.0012410688, SE = 0.0004288376, *p* = 0.005086891), whereas the weighted median estimate was positive but not statistically significant (β = 0.00059, *p* = 0.173). The MR-Egger intercept was not significant, indicating no strong evidence of directional horizontal pleiotropy in this dataset. The forest plot summarizes the effect estimates and 95% confidence intervals obtained using the three MR methods (Figure 4B). In the scatter plot, the SNP effects on LDL and AD showed an overall positive trend, although the magnitude of the estimated effect was small (Figure 4C). In addition, leave-one-out analysis did not identify any single SNP that disproportionately drove the overall IVW estimate (Figure 4D). Taken together, these findings suggest that higher genetically predicted LDL levels may be associated with increased AD risk. However, the effect size was modest and the results should be interpreted with caution.

The MR analysis employs genetic variants as instrumental variables for LDL levels, yielding insights into population-level causality that are orthogonal to the molecular docking and network pharmacology results.

In summary, the MR analysis provides genetic evidence that lifelong exposure to higher LDL levels increases AD risk, supporting the classification of lipid metabolism disorders as a modifiable risk factor for Alzheimer’s disease.

### 2.6. Computational Analysis of Lonicera caerulea Targets Shared Between AD and LMDs

**Figure 5:**
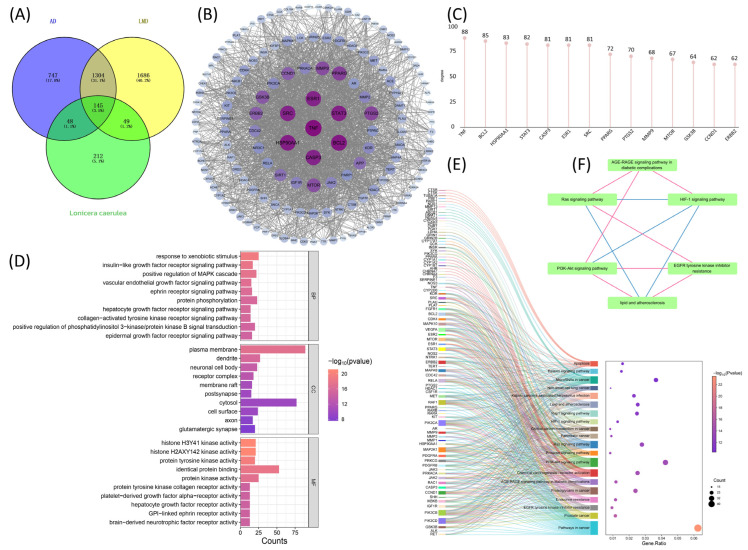
Analysis of the intervention effect of *Lonicera caerulea* on AD and LMDs. (**A**) The intersection between the targets of *Lonicera caerulea* and co-targets of AD and LMDs. (**B**) The PPI network of prospective targets. Rotundity represented proteins, the color and size of the targets reflected their degree values such that darker color and larger diameter indicated higher degree values, and the lines represented protein interactions. (**C**) The bar chart of PPI degree values showed top seven targets were TNF, BCL2, HSP90AA1, STAT3, CASP3, ESR1, and SRC (**D**) The GO analysis of the overlapping targets (molecular function, cellular component, biological process). (**E**) The Sankey diagram with bubble plot for KEGG enrichment analysis. (**F**) Pink lines represent activating effects and blue lines represent regulating effects. EGFR tyrosine kinase inhibitor resistance activated PI3K-Akt signaling pathway and Ras signaling pathway; PI3K-Akt signaling pathway and Ras signaling pathway regulated HIF-1 signaling pathway; AGE-RAGE signaling pathway in diabetic complications activated PI3K-Akt signaling pathway, Ras signaling pathway and HIF-1 signaling pathway.

The 454 targets of *Lonicera caerulea*, 2244 targets of Alzheimer’s disease, and 3184 targets of lipid metabolism disorders were intersected, resulting in 145 common targets (Figure 5A). Based on the STRING database and Cytoscape, a protein–protein interaction network diagram was generated (Figure 5B). The results showed that the PPI network compromised 145 nodes and 2018 edges. The hub targets included TNF, BCL2, HSP90AA1, STAT3, CASP3, SRC, and ESR1 (Figure 5C).

To further explore the potential relationships among key targets and enriched pathways, we carried out GO and KEGG enrichment analysis using the 145 overlapping targets.

In GO enrichment analysis, it was revealed that the targets related to *Lonicera caerulea*, AD, and LMDs were primarily associated with “response to xenobiotic stimulus”, “insulin-like growth factor receptor signaling pathway”, “positive regulation of MAPK cascade” in BPs; “plasma membrane”, “dendrite”, “neuronal cell body” in CCs; and “histone H3Y41 kinase activity”, “identical protein binding”, “protein tyrosine kinase activity” in MFs (Figure 5D).

Since ESR1 is mainly involved in cancer-related pathways, we did not investigate it in KEGG enrichment analysis. Significantly, we found that the first six of the above hub targets were statistically enriched in the lipid and atherosclerosis pathway (Figure 5E). Additionally, five KEGG-enriched pathways—including EGFR tyrosine kinase inhibitor resistance, PI3K–Akt signaling pathway, AGE–RAGE signaling pathway in diabetic complications, HIF-1 signaling pathway, and Ras signaling pathway—exhibited substantial crosstalk at the levels of genes, transcription factors, and metabolic enzymes. This interconnected network collectively contributes to aberrant lipid accumulation, inflammatory activation, and plaque instability, thereby constituting the potentially interconnected pathways of lipid and atherosclerosis (Figure 5F). These analyses point to possible functional links. Whether they hold true in vivo is a different question that needs further experiments to pin down their biological significance.

### 2.7. The Component-Target-Disease Diagram Reveals the Multi-Target and Multi-Pathway Interaction Mechanisms

**Figure 6:**
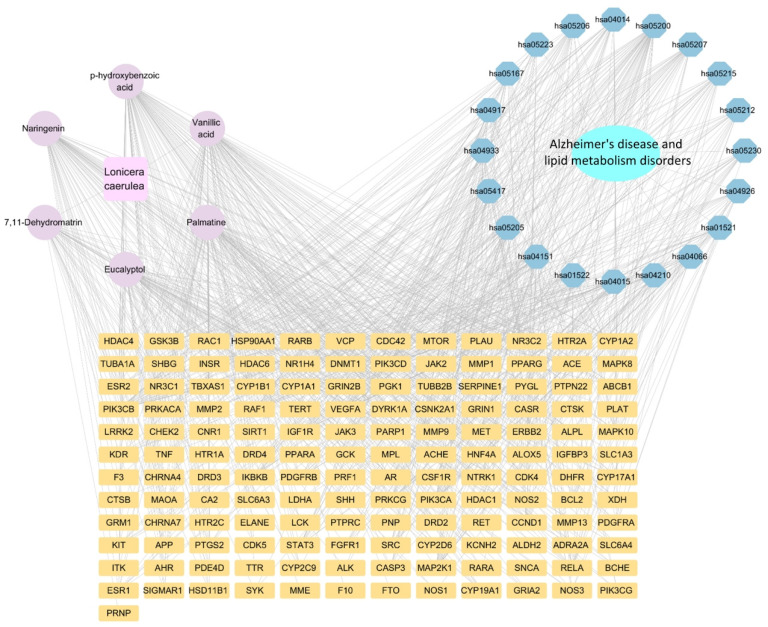
Component–target–disease network delineating the interactions between the six core components of *Lonicera caerulea* and the top 20 KEGG pathways associated with AD and LMDs. Ellipse represents AD and LMDs, circle represents the component of *Lonicera caerulea*, square represents protein targets, and hexagon represents the pathway.

To support our hypothesis, we used Cytoscape version 3.10.2 to construct a CTD network analysis based on the correlation analysis among *Lonicera caerulea*, Alzheimer’s disease and Lipid metabolism disorder (Figure 6). The results showed that the main active components of *Lonicera caerulea* acting on AD and LMDs were Eucalyptol, p-hydroxybenzoic acid, Vanillic acid, Naringenin, Palmatine, and 7,11-Dehydromatrin. Chemicals with relatively high connectivity exhibited a tendency to interact with most targets in the network, thus potentially serving as the primary targets for *Lonicera caerulea*’s effects on AD and lipid metabolism. Similarly, proteins TNF, BCL2, HSP90AA1, STAT3, CASP3, SRC, and ESR1 exhibited high degree values in the network, indicating their extensive interactions with multiple components. In addition, expression products of enriched targets participated in the constitution and functional execution of some KEGG pathways related to AD and LMDs, such as Lipid and atherosclerosis, AGE-RAGE signaling pathway in diabetic complications, Endocrine resistance, PI3K-Akt signaling pathway, Rap1 signaling pathway, and Apoptosis. These findings collectively suggested that *Lonicera caerulea* may exert its intervention effects by regulating multiple targets and pathways, leading to complex pharmacological changes that still need to be validated.

### 2.8. Molecular Docking Results

#### 2.8.1. Redocking Results

To assess the reliability of the molecular docking method, we performed re-docking calculations using the co-crystallization ligands retrieved from the respective PDB file entries of each protein. All parameter settings for the docking process (including the size and position of the docking grid box) were kept exactly the same as those used in the exploratory docking phase. The redocking results are presented in Table 3.

Under large-scale conditions where the grid box volume exceeded 27,000 Å^3^, the RMSD values for redocking of both the HSP90AA1 and BCL2 systems were still within 2.0 Å, preliminarily indicating that the molecular docking method employed exhibits good robustness. The redocking RMSD values for TNF (8.94 Å) and CASP3 (9.24 Å) exceeded the conventional 2.0 Å threshold, suggesting that the docking results for these targets may not be as reliable as those for other systems. This discrepancy likely arises from pronounced induced-fit effects or substantial conformational changes within the shallow binding pockets, which the rigid-receptor docking protocol failed to capture adequately. Consequently, the docking poses for these two targets should be interpreted with greater caution and are considered preliminary hypotheses requiring further experimental validation. The co-crystal ligand KQV in STAT3 does not bind to the typical SH2 domain, making re-docking validation of limited significance.

#### 2.8.2. Exploratory Docking Results

**Figure 7:**
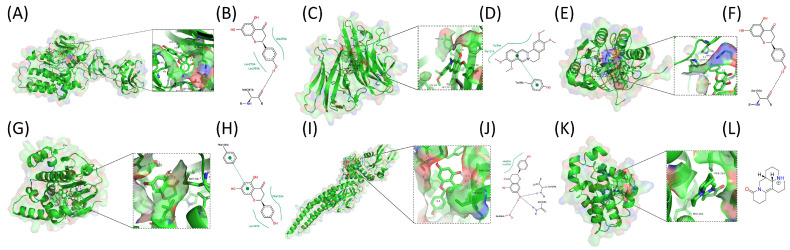
The 2-dimensional and 3-dimensional map of the binding sites between small molecule compounds of *Lonicera caerulea* and target proteins. (**A**,**B**) (Binding outcomes of SRC with Naringenin) represent the same set of molecular docking output in both two-dimensional and three-dimensional forms. (**A**) presents the spatial binding state of the small molecule (shown as a green stick model) within the protein’s active pocket in three dimensions. A magnified view clearly illustrates the binding mode of the small molecule to the protein, with Nitrogen atom depicted as blue stick models. Oxygen atom depicted as red stick models. Sulfur atoms are indicated by yellow stick models. (**B**) displays the interactions between the small molecule entity and key amino acid residues of the protein in two dimensions, including hydrogen bonds and hydrophobic interactions, among others. (**C**,**D**) (Binding outcomes of TNF with Palmatine), (**E**,**F**) (Binding outcomes of CASP3 with Naringenin), (**G**,**H**) (Binding outcomes of HSP90AA1 with Naringenin), (**I**,**J**) (Binding outcomes of STAT3 with Naringenin), (**K**,**L**) (Binding outcomes of BCL2 with 7,11-Dehydromatrin) correspond to six sets of molecular docking results, respectively. The binding modes are consistent with structure–activity relationship (SAR) data and account for their high binding affinity.

Based on the above analysis, we ultimately identified six representative protein receptors: TNF, BCL2, HSP90AA1, STAT3, CASP3 and SRC. Molecular docking was performed between these receptors and six active ingredients from *Lonicera caerulea*. The docking mode is shown in Figure 7 and Figure S2. Small molecules primarily bind to proteins through hydrogen bonding, pi-pi interaction, and hydrophobic interactions. In Table 4, the optimal docking results for the active components of *Lonicera caerulea* and the major protein receptors were determined by comparing the docking binding energies and the number of hydrogen bonds and pi-pi interactions. In the SRC system, the binding mode of the ligand Naringenin exhibits a typical hydrogen bond directed anchoring characteristic: the two ends of the small molecule formed hydrogen bonds with the deep and shallow regions of the binding pocket respectively, thereby ensuring a unique binding orientation. In contrast, within the TNF, CASP3, and STAT3 systems, the ligand tends to form a hydrogen bond network only on one side of the small molecule to mediate binding.

Notably, Naringenin does not form water-mediated hydrogen bonds via conserved water molecules within the HSP90AA1 binding pocket, and 7,11-Dehydromatrin forms no hydrogen bonds or pi-pi interactions.

### 2.9. Molecular Dynamics Simulation Results

**Figure 8:**
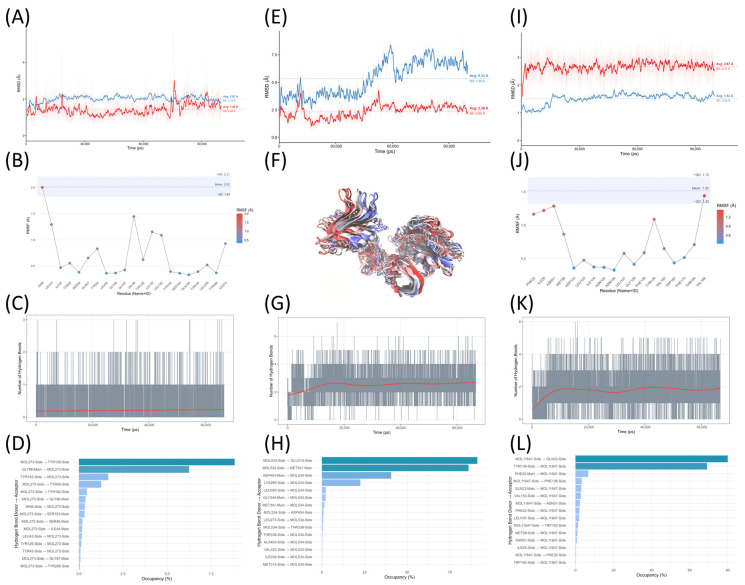
Analysis of the stability and interactions from molecular dynamics simulations of three complex systems. (**A**) Plot of the root-mean-square deviation (RMSD) over time for the Palmatine-TNF system, where the blue curve represents the intrinsic motion of the protein backbone carbon atoms, and the red curve represents the motion of the ligand relative to the protein backbone carbon atoms. The thick lines represent the average of every 50 adjacent frames, which is used to suppress thermal noise; the thin lines in the background represent the raw data. (**B**) In Palmatine-TNF system, the root-mean-square fluctuation (RMSF) distribution of each residue in the ligand binding pocket region, and the color of data points from blue to red indicates that the RMSF value is from low to high. The blue band at the top shows the mean and standard deviation of RMSD of protein skeleton carbon atoms. (**C**) The number of hydrogen bonds between protein and ligand in Palmatine-TNF system changes with simulation time, and the red curve is the statistical fitting result based on the generalized additive model (GAM). (**D**) The time occupation of hydrogen bonds between various residues and ligands in Palmatine-TNF system. Bar colors represent occupancy, with darker shades indicating higher occupancy. (**E**) RMSD evolution curve over time for the Naringenin-SRC system. (**F**) Conformation overlay of the SRC protein during the simulation; the protein is depicted in cartoon form and colored according to simulation time (red-white-blue corresponds to the progression of the simulation from early to late stages). (**G**) Changes in the number of protein-ligand hydrogen bonds in the Naringenin-SRC system over simulation time. (**H**) The time distribution of hydrogen bond formation between residues and ligands in the Naringenin-SRC system. (**I**) RMSD evolution curves for the Naringenin-HSP90AA1 system over time. (**J**) RMSF distribution of each residue in the ligand binding pocket region in Naringenin-HSP90AA1 system. (**K**) The number of hydrogen bonds between protein and ligand in Naringenin-HSP90AA1 system changed with simulation time. (**L**) The time occupation of hydrogen bond between each residue and ligand in Naringenin-HSP90AA1 system.

In order to further verify the credibility of molecular docking results, we selected Palmatine-TNF, Naringenin-SRC and Naringenin-HSP90AA1 three complex systems to conduct molecular dynamics simulations up to 100 ns, respectively. The results are visualized in Figure 8, and MD results of other complexes are shown in Figure S3.

The simulation results showed that in Palmatine-TNF, Naringenin-SRC and Naringenin-HSP90AA1 systems, the RMSD of ligand relative to the carbon atom of protein skeleton could be maintained at about 1.8 Å, and the fluctuation range was controlled within 0.5 Å, showing good binding stability. In particular, the protein backbone carbon atoms in the Palmatine-TNF and Naringenin-HSP90AA1 systems exhibit an RMSD of approximately 1.7 Å and a fluctuation range of about 0.2 Å, indicating a stable conformational state. For the Naringenin-SRC system, the RMSD of protein skeleton carbon atoms was significantly adjusted around 40 ns; trajectory dynamic analysis showed that the change was mainly due to the aggregation trend between two protein domains connected by two peptide chains (Figure 8F).

The results of the hydrogen bonding analysis show that the three systems—Palmatine-TNF, Naringenin-SRC, and Naringenin-HSP90AA1—form 1, 3, and 2 stable hydrogen bonds, respectively. In the Palmatine-TNF system, no stable hydrogen bond donor–acceptor pair was observed, indicating that the hydrogen bond played only a secondary role in this binding mode. In contrast, the Naringenin-SRC system contains two sets of highly stable hydrogen bond donor–acceptor pairs, with occupancy rates of 91.07% and 85.91%, respectively, over the 100 ns simulation period; the Naringenin-HSP90AA1 system also has two sets of stable hydrogen bond donor–acceptor pairs, with occupancy rates of 79.94% and 69.05%, respectively. These results suggest that the ligand binding modes have clear hydrogen bond anchoring characteristics, which is helpful to maintain the structural stability of the complexes.

## 3. Discussion

The compound list of *Lonicera caerulea* was compiled from literature and preliminary LC-MS/MS data (Table S1), though most compounds lacked full MS/MS validation or quantitative confirmation, which may limit reproducibility. We are currently conducting comprehensive characterization of *Lonicera caerulea* components for separate publication. These results should therefore be considered exploratory, requiring validation with fully characterized standards. Despite the limitation, the current dataset provides a useful basis for hypothesis-generating analyses. Our integrated network pharmacology approach identified six blood-brain-barrier-permeable, bioactive components from *Lonicera caerulea*, with 454 predicted protein targets. Enrichment analysis of these targets revealed an overrepresentation of terms related to GPCRs and serotonin receptors. Given the pivotal roles of these pathways in neuromodulation and systemic metabolism, this finding directed our investigation toward neuropsychiatric and metabolic disorders, specifically Alzheimer’s disease (AD) and lipid metabolism disorders (LMDs). We computationally evaluated the potential of *Lonicera caerulea* for concurrent intervention in these comorbid conditions.

PPI network analysis of the predicted targets identified seven core hub targets: TNF, SRC, STAT3, ESR1, BCL2, HSP90AA1, and CASP3. These proteins have been previously implicated in processes relevant to neuroinflammation, apoptosis, and cellular stress responses. For example, TNF mediates neuroinflammation, promoting neurotoxicity and neurodegeneration via pro-inflammatory cytokine signaling [33]. In Alzheimer’s disease, HSP90AA1 is abnormally expressed in the entorhinal cortex, disrupting the functions of microglia and astrocytes, impairing synaptic homeostasis and exacerbating Aβ pathology [34]. Its dysfunction also contributes to APOE4-mediated gliosis, tau pathology and neuronal loss [35]. SRC is elevated in neurons and interacts with PYK2 to modulate signaling and inflammatory pathways, contributing to Aβ toxicity and tau phosphorylation in Alzheimer’s disease [36]. This process concurrently activates microglia to release pro-inflammatory factors (e.g., TNF-α, IL-1β), establishing a pernicious cycle of oxidative stress and inflammation that further exacerbates neuronal apoptosis [37]. However, it should be noted that these hub targets were identified solely based on topological features of the PPI network. While such metrics can highlight potentially important nodes, they do not necessarily reflect actual biological significance or causal roles in disease processes, and therefore require further validation.

To further explore the potential relationship between AD and LMDs, we performed comorbidity analysis and two-sample Mendelian randomization (MR). The comorbidity analysis identified shared core targets between the two diseases, with a 40% overlap with the core targets of *Lonicera caerulea*. This substantial overlap generates the hypothesis that a shared mechanism might underlie a dual intervention. Furthermore, the MR analysis provided genetic evidence supporting low-density lipoprotein (LDL) as a potential causal risk factor for AD, thereby reinforcing the epidemiological link between AD and LMDs and highlighting a modifiable risk axis [9].

Based on this, we intersected the targets of *Lonicera caerulea*, AD, and LMDs for subsequent enrichment and network analyses. Notably, the top-ranked hub targets identified in this subset were consistent with those obtained from separate analysis of *Lonicera caerulea*. This remarkable overlap implies that these seven proteins may represent not only key targets of *Lonicera caerulea* but also the central hubs between AD and LMDs. While this overlap may suggest network-level convergence, it should be noted that such consistency may also reflect inherent biases in network topology and database annotations. In the top 20 KEGG analyses ranked by *p*-value, we found that six of the top seven targets (TNF, SRC, STAT3, BCL2, HSP90AA1 and CASP3) in the PPI were concentrated in the lipids and atherosclerosis pathway. Therefore, these six targets were prioritized for further computational exploration. In addition, the other five significant enrichment pathways in KEGG are also involved lipid and atherosclerosis, implying the complexity and comprehensiveness of potential regulatory mechanisms. Lipid metabolism disorders initiate a cascade of pathological alterations—including inflammation, smooth muscle cell migration and proliferation, and necrotic core formation—that collectively accelerate atherosclerotic progression [38]. Notably, atherosclerosis and AD share common risk factors, such as obesity, dyslipidemia, and diabetes, which promote the development of both intracranial and extracranial atherosclerosis. This vascular pathology, in turn, precipitates cerebral hypoxia and hypoperfusion. Under hypoxic conditions, enhanced β-/γ-secretase activity drives aberrant cleavage of amyloid precursor protein (APP), resulting in excessive β-amyloid production and subsequent amyloid plaque deposition—a hallmark pathological feature of AD. Simultaneously, cerebral hypoperfusion induces neuronal energy crisis, triggering neurodegenerative cascades that further propel AD pathogenesis [39]. KEGG analysis implicates the lipid and atherosclerosis pathway in the molecular overlap between AD and LMDs, while MR data point to a potential causal link between LDL and AD, highlighting the potential of lipid dysregulation in neurodegenerative pathology. Although these two lines of evidence cannot directly corroborate each other, they collectively point towards lipid biology as the priority direction for validation.

To further verify the binding potential between the core target proteins and the active small molecules, molecular docking and molecular dynamics simulations were performed. Molecular docking results suggested that the six candidate compounds bind to their respective targets mainly through hydrogen bonds, π-π stacking, and van der Waals interactions. Re-docking validations, though not ideal for all systems, suggested the overall rationality of the docking method. Collectively, the docking results indicate that the screened active small molecules exhibit good binding performance with all six core target proteins under modeled conditions.

Molecular dynamics simulations (100 ns) further supported the binding stability of most complexes, with no significant conformational deviations from the initial docking structures observed in most systems. The domain compaction seen in the SRC-Naringenin system is attributable to a known systematic bias of the force field rather than physical instability. Stable hydrogen bond networks were observed in multiple systems, indicating synergistic anchoring of ligands by both polar and nonpolar interactions. Together, these simulation results provide supporting evidence that the active components of *Lonicera caerulea* may have binding potential toward these targets, intimating possible interactions at the molecular level, and experiments need to be done in further research.

Based on the initial screening of component effectiveness, network analysis to identify core targets, and docking and kinetic validation of binding stability, Naringenin and Palmatine were selected as representative compounds for further discussion. Previous studies have reported that Naringenin may influence cholesterol metabolism and vascular function [40,41], while Palmatine has been associated with anti-inflammatory effects [42]. Meanwhile, both Palmatine and Naringenin can remold gut microbiota to regulate lipid metabolism via lowering low density lipoprotein cholesterol (LDL-C) level [41,42,43]. These independent findings align well with the lipid-related pathways identified in our analysis, reinforcing the relevance of the active phytoconstituents to these pathways through binding to their targets.

Existing research has shown that Naringenin, a dihydroflavonoid compound, exerts multifaceted anti-AD effects through multiple pathways: it can reduce Aβ generation and deposition by down-regulating BACE1 expression and restraining amyloidogenic APP processing; attenuate neuroinflammation via MAPK signaling pathway inhibition, thereby reducing microglial and astrocytic activation; modulate the PI3K/Akt/GSK3β pathway to decrease tau hyperphosphorylation; and suppress NF-κB-mediated p65 nuclear translocation, consequently reducing pro-inflammatory factor production (e.g., TNF-α and IL-1β) [22,44]. Additionally, Palmatine can cross the blood–brain barrier, improve cognitive impairment, reverse proteomic abnormalities in the hippocampus and cerebellum of mice, and thus emerges as a potential candidate drug for the treatment of Alzheimer’s disease [19]. However, our study implies that Naringenin and Palmatine indirectly affect AD by influencing the lipid and atherosclerosis pathway. These findings not only expand the putative mechanisms by which Naringenin and Palmatine may be involved in AD, but also prompts that *Lonicera caerulea* is likely to be engaged in AD through both direct and indirect mechanisms.

**Figure 9:**
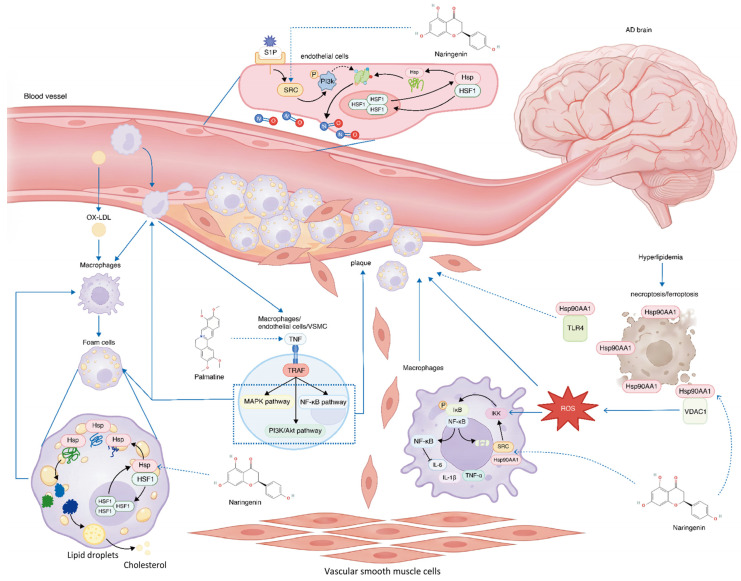
The schematic diagram showing that the core components of *Lonicera caerulea* (Naringenin and Palmatine) ameliorate atherosclerosis by regulating macrophages, endothelial cells and vascular smooth muscle cells (VSMCs), a process that may be relevant to processes associated with AD and LMDs. Solid arrows indicate the activation or promotion of biological processes, while T-shaped arrows indicate the inhibitory effect on signaling pathways. The dashed arrows represent regulatory effects (including both indirect effects and the predicted regulatory effects of active ingredients). In this figure, key proteins in foam cells fold correctly after HSP regulation to promote cholesterol outflow and maintain lipid homeostasis. Rectangular structures in the macrophage nucleus are κB binding sites on DNA. NF-κB anchors to these sites and initiates the transcription and expression of inflammation-related target genes.

From a systems-level perspective, our data suggests that the intervention effects of *Lonicera caerulea* may be mediated through key hub targets, including HSP90AA1, TNF, and SRC (Figure 9). Importantly, these targets not only are implicated in classical AD pathology—such as Aβ accumulation, tau hyperphosphorylation, and synaptic dysfunction—but also play critical roles in lipid and atherosclerosis by modulating PI3K-AKT, NF-κB, MAPK, and other signaling pathways [45,46,47]. It is reported that they jointly maintain normal cell physiology, enhance NO production and cholesterol efflux [45,48,49], and inhibit apoptosis as well as extracellular plaque formation induced by factors such as OX-LDL [45,46]. This dual involvement supports the notion that AD and LMDs are not independent conditions but are biologically interconnected through shared molecular networks. Compared with previous studies which examine these targets in single-disease contexts, our work provides a more integrative perspective, implying that simultaneous modulation of apoptosis, protein folding, and lipid homeostasis may produce synergistic therapeutic effects.

In this study, we focused on the effect of BBB-permeable components in *Lonicera caerulea* on AD and LMDs. This restriction likely underestimates peripheral mechanisms now implicated in AD pathophysiology, particularly lipid metabolism and systemic inflammation. Several excluded non-BBB-permeable compounds, such as cyanidin, quinic acid, and catechin, have been reported to modulate systemic lipid metabolism and inflammatory responses [50,51]. Future studies should expand beyond BBB-permeable constituents to capture peripheral mechanisms, particularly those related to systemic metabolism. In addition, a key limitation of this study is the reliance on enrichment-based inference to prioritize pathways. Although the lipid and atherosclerosis pathway was consistently identified across analyses, no pathway-specific experimental validation was performed, and enrichment results may be influenced by database bias and gene annotation density. In the future, the integration of multi-omics approaches may provide a more comprehensive understanding of the underlying mechanisms.

## 4. Materials and Methods

### 4.1. Target Screening of Lonicera caerulea Components

*Lonicera caerulea* components were identified through literature mining and preliminary LC-MS/MS. Then, TCMSP (https://tcmsp-e.com/tcmsp.php, accessed on 3 April 2025) was employed to screen the potential active components of *Lonicera caerulea*, according to the conditions of oral bio-availability (OB) ≥ 30% and blood–brain barrier (BBB) ≥ −0.3. Meanwhile, SMILES for each active component was retrieved from the PubChem database (https://pubchem.ncbi.nlm.nih.gov/, accessed on 5 April 2025). We further evaluated these components for the Rule of Five (RO5) using SwissADME (http://www.swissadme.ch/, accessed on 6 April 2025) and conducted toxicological assessments via ProTox 3.0 (https://tox-new.charite.de/protox_II/, accessed on 6 April 2025). After completing all the above operations, the core active components were eventually determined. Then we obtained their corresponding targets (probability > 0) from Swiss Target Prediction (http://www.swisstargetprediction.ch/, accessed on 20 April 2025) and TargetNet (accessed on 21 April 2025). Finally, we used the Uniprot database (https://www.Uniprot.org/, accessed on 23 April 2025) to convert the obtained Uniprot ID of the targets and gained the corresponding gene names.

### 4.2. GO Enrichment and KEGG Pathway Analysis

To elucidate the functional and pathway context of the relevant targets, Gene Ontology (GO) and Kyoto Encyclopedia of Genes and Genomes (KEGG) pathway enrichment analysis were performed. The GO analysis provides systematic functional annotations across three key aspects: biological processes (BPs), cellular components (CCs), and molecular functions (MFs), offering a comprehensive understanding of the biological roles of the targets. Concurrently, KEGG pathway enrichment analysis was conducted to identify condition-specific metabolic pathways and functional modules significantly associated with the targets, thereby uncovering their involvement in key signaling and biological processes. These analyses were carried out using the DAVID database (https://davidbioinformatics.nih.gov/, accessed on 9 May 2025) and the Weishengxin platform (accessed on 11 May 2025). The results of analysis deepened understanding of these targets’ functional enrichment and pathway associations, offering insights into the pharmacological potential of *Lonicera caerulea* and potential disease-related therapeutic targets.

### 4.3. The Protein–Protein Interaction Network Analysis

Targets corresponding to *Lonicera caerulea* were imported into the STRING database (https://cn.string-db.org, accessed on 14 May 2025) for the identification and construction of protein–protein interaction (PPI) networks, selecting ‘*Homo sapiens*’ and choosing targets with a fitting score above 0.9. After that, we got a visualized net with Cytoscape 3.10.2. In the composite target network, nodes denote components or targets, and edges represent their connections. By using cytoNCA plugin, we conducted a more in-depth analysis of the obtained results.

### 4.4. Collection of Predicted Targets for AD and LMDs

Target genes associated with Alzheimer’s disease and lipid metabolism disorders were searched through the GeneCards (https://www.genecards.org/, accessed on 17 May 2025), OMIM (https://www.omim.org/, accessed on 18 May 2025), CTD (accessed on 20 May 2025) and TTD (accessed on 20 May 2025). The top 2000 targets with the highest scores were chosen in GeneCards. All human-associated targets were selected from OMIM. In addition, genes with direct evidence in CTD and all targets in TTD were also included. Finally, we combined the targets obtained from four databases for the two keywords mentioned above, thus resulting in target gene sets for AD and LMDs.

### 4.5. Analysis of Co-Expressed Targets Between Alzheimer’s Disease and Lipid Metabolism Disorders

Co-expressed targets related to AD and LMDs were imported into String for the PPI networks and then into Cytoscape for further topological analysis. Subsequently, four metrics from CytoHubba were applied: Degree and EPC for the top 10 targets, and Closeness and Betweenness for the top 20 targets (each metric independently). And then, we intersected all of them to determine the final hub targets.

### 4.6. Mendelian Randomization (MR) Analysis

#### 4.6.1. GWAS Summary Statistics of LDL and AD

The LDL data (ebi-a-GCST90092961) and the AD data (ieu-b-5067) were derived from summary statistics of two large-scale genome-wide association studies (GWAS) in the OpenGWAS database (accessed on 2 June 2025). The former was based on 115,082 individuals of European ancestry (mixed sex) and included 11,590,399 single nucleotide polymorphisms (SNPs). The latter was based on 488,285 participants of European ancestry (both males and females) from the UK Biobank (accessed on 3 June 2025). Within this cohort, 12,321,875 SNPs obtained from 954 AD patients were analyzed using the BOLT-LMM analysis method.

#### 4.6.2. Construction of Instrumental Variables for MR Analysis

We selected significant and independent SNPs from the LDL dataset as instrumental variables (IVs) for forward MR analysis based on the following criteria. SNPs significantly associated with LDL levels were screened at *p* < 5 × 10^−8^. Linkage disequilibrium (LD) clustering was performed using the 1000 Genomes European reference panel (window size = 10,000 kb, r^2^ < 0.001), retaining the index SNP with the lowest *p*-value within each window. To ensure IV robustness, each instrument was assessed by calculating the F-statistic, confirming values exceeding 10.

#### 4.6.3. Implementation of MR Analysis and Sensitivity Analysis

The study employed the ‘TwoSampleMR’ R package (v0.6.15) for two-sample MR analysis between LDL and AD. The inverse variance weighting (IVW) method was employed as the primary analysis approach. Results were discussed in conjunction with MR-Egger regression and weighted median methods. Level multi-effectivity was assessed via the MR-Egger intercept, while heterogeneity was quantified using Cochran’s Q statistic. Additionally, leave-one-out analysis was conducted as a sensitivity analysis to ensure robustness of findings. Statistical significance was set at α = 0.05.

### 4.7. Diseases and Lonicera caerulea GO, KEGG Enrichment Analysis, and PPI Network Construction

To identify the effective targets of *Lonicera caerulea* in intervening AD and LMDs, we used a Venn diagram to find their intersection. Using the String database, a PPI network was constructed from the obtained effective targets, and its visualization was accomplished with Cytoscape 3.10.2. Also, the GO and KEGG enrichment analysis were performed through importing the effective targets into the DAVID platform.

### 4.8. Construction of the “Component-Target-Disease (CTD)” Network

The obtained active ingredients, enriched pathways and screened genes are integrated into a type file. The genes corresponding to each active ingredient and key pathway, the pathways corresponding to disease and the active ingredients corresponding to *Lonicera caerulea*, are listed one by one to form a network file. The type file and network file are imported into Cytoscape (3.10.2) for network visualization.

### 4.9. Molecular Docking Protocol

#### 4.9.1. Exploratory Docking

The core protein receptors in the PPI network were submitted to the RCSB PDB database (https://www.rcsb.org/, accessed on 3 June 2025) for retrieval. High-quality X-ray crystal structures were selected as the reference conformations of each target protein, and the corresponding docking pockets were defined based on the known active sites or conventional small-molecule binding regions of each protein. Relevant details are summarized in Table 5, with grid box center coordinates and dimensions provided in Tables S3–S5. During protein preparation, free water molecules, co-crystallized ligands, and solvents were removed, except for four conserved water molecules in the binding pocket of HSP90AA1. For residues with multiple conformations, only the highest-occupancy form was retained. Subsequently, protonation states were assigned according to physiological pH, followed by the addition of Gasteiger charges. The 3D structural information of small-molecule ligands were obtained from the PubChem database (https://pubchem.ncbi.nlm.nih.gov/, accessed on 3 June 2025). During ligand preparation, the protonation states of the ligands were uniformly adjusted to pH 7.4 ± 2.0, and Gasteiger charges were also assigned. Molecular docking was performed using AutoDock Vina 1.1.2, with exhaustiveness set to 128, which is 16 times the default value, to improve conformational sampling within a search space exceeding 27,000 Å^3^. Results are visualized by PyMoL 2.6.2 and ProteinPlus (https://proteins.plus, accessed on 11 July 2025). The affinity is calculated by scoring functions [52].

#### 4.9.2. Redocking

Redocking was performed using co-crystallized ligands retrieved from the PDB file. The reliability of the molecular docking method was evaluated by calculating the root-mean-square deviation (RMSD) between the docked conformations and the experimental structures. Notably, the same grid box parameters and exhaustiveness settings as in the initial docking were applied. A smaller grid box tailored to the co-crystallized ligand was intentionally avoided, as such conditions primarily evaluate the intrinsic performance of AutoDock Vina rather than its robustness under practical, large-scale docking scenarios.

### 4.10. Molecular Dynamics Simulation

Protein pretreatment: the PDBFixer tool is used to complement the missing heavy atoms in the protein structure, and adjust the protonation state of amino acid residues according to physiological pH conditions. Next, use the pdb2gmx module in the GROMACS 2026 software package to generate a protein topology file. For the SRC structure, due to the presence of missing loop segments, we first used the Modeller program to complete it through homology modeling. Pretreatment of small molecule ligands: the protonation state of small molecule ligands was uniformly adjusted to pH 7.4 ± 2.0. Geometric optimization and frequency analysis of small molecules were performed using the Gaussian program at the HF/6-31G* level, and Restrained Electrostatic Potential (RESP) charges were calculated using the Multiwfn 2026.4.10 software based on these results [53]. The small-molecule topology files were generated by the Sobtop 2026.1.16 [54], and the force field parameters were defined based on the GAFF2 force field.

All molecular dynamics simulations were performed using the GROMACS 2026. One package. Proteins were described by AMBER19SB force field, small molecule ligands were described by GAFF2 force field, and solvent water molecules were represented by the TIP3P model. The simulated box was based on the geometric center of the protein, extending 1.0 nm in all directions, and the widest dimension was used to construct the cubic box. After filling the system with TIP3P water molecules, Na^+^ and Cl^−^ ions were added to adjust the salt concentration to physiological levels (0.15 mol/L). The system temperature was controlled using the V-rescale thermostat. To avoid the “Flying Ice Cube” effect associated with this thermostat, the protein–ligand complex and the remaining components (water molecules and ions) were grouped separately, with each group independently maintained at a physiological temperature of 310 K. During the energy minimization stage, all atoms in the system are relaxed until the maximum force acting on any atom falls below 100 kJ·mol^−1^·nm^−1^; for the SRC system, which exhibits potential unreasonable contacts following homogeneous mode-based construction, the preliminary convergence criterion is relaxed to below 300 kJ·mol^−1^·nm^−1^. On this basis, a position-restricted potential of 1000 kJ·mol^−1^·nm^−2^ was applied to the protein–ligand complex, and a 100 ps restricted molecular dynamics simulation was performed with a 1 fs integration step to allow for the full relaxation of water molecules and ions. In the final stage of production dynamics a 2 fs integration step size was used, with 100 ns of sampling under unconstrained conditions; the system’s conformation was recorded every 10 ps to ensure sufficient statistical independence between trajectory frames.

Before the subsequent analysis, the simulated trajectory is processed by time continuity correction, spatial continuity sorting and rotation elimination. The root-mean-square deviation (RMSD) of the evolution of protein skeleton atoms over time was calculated by the built-in tool gmx rms of GROMACS; hydrogen bond analysis is completed with the help of the hydrogen bonds plug-in in VMD1.9.4 software; and the root-mean-square fluctuation (RMSF) of each amino acid residue was calculated by gmx rmsf. All visualizations are based on R.

## 5. Conclusions

To sum up, this study employed network pharmacology and computational modeling to explore the potential multi-component, multi-pathway, and multi-target mechanisms by which *Lonicera caerulea* may influence Alzheimer’s disease and lipid metabolism disorders. The analyses predict that the principal components of *Lonicera caerulea*, Naringenin and Palmatine, can bind to targets such as HSP90AA1, SRC, and TNF, which are implicated in the lipid and atherosclerosis pathway. To some extent, these interactions imply a presumable role in improving the cerebral vascular microenvironment, thereby potentially contributing to the intervention in the progression of Alzheimer’s disease and lipid metabolism disorders.

## Figures and Tables

**Table 1:** Screening of the core components of *Lonicera caerulea*.

MolID	Compound	OB/%	BBB	RO5
MOL005970	Eucalyptol	39.72	0.62	qualified
MOL000103	p-hydroxybenzoic acid	30.14	0.20	qualified
MOL000114	Vanillic acid	35.47	0.09	qualified
MOL004328	Naringenin	59.29	0.03	qualified
MOL000785	Palmatine	64.60	0.37	qualified
MOL006583	7,11-Dehydromatrin	44.42	1.12	qualified

OB%: bio-availability BBB: blood–brain barrier RO5: Rule of Five.

**Table 2:** Estimated values (β), Standard Errors (SE), and *p*-values for the three MR methods.

Methods	Estimated Values (β)	Standard Errors (SE)	*p*-Values
Inverse variance weighted	0.0007458163	0.0002876124	0.009510689
MR Egger	0.0012410688	0.0004288376	0.005086891
Weighted median	0.0005880844	0.0004319288	0.173345957

**Table 3:** Docking RMSD value of each protein cocrystal ligand.

PDB ID	Protein Name	RMSD (Å)
1Y57	SRC	3.84
2AZ5	TNF	8.94
3KJF	CASP3	9.24
3O0I	HSP90AA1	1.87
6NJS	STAT3	N/A
6O0K	BCL2	1.68

**Table 4:** Statistics of the small molecule with the best affinity in each protein, including the number of hydrogen bonds and RMSD.

Protein	Best Ligand	Affinity (kcal/mol)	HBonds
SRC	Naringenin	−8.1	2
TNF	Palmatine	−7.8	1
CASP3	Naringenin	−6.9	3
HSP90AA1	Naringenin	−9.0	0
STAT3	Naringenin	−7.3	3
BCL2	7,11-Dehydromatrin	−7.3	0

**Table 5:** PDB ID of the selected structure of each protein and the defined docking pocket position.

Protein	PDB ID	Docking Pocket
SRC	1Y57	ATP-binding site
TNF	2AZ5	trimeric interface
CASP-3	3KJF	S1-S4 subsites
HSP90AA1	3O0I	ATP-binding pocket
STAT3	6NJS	SH2 domain
BCL-2	6O0K	BH3-binding groove

Note: The definition of CASP3 binding pocket covers all four sub sites from S1 to S4.

## Data Availability

The original contributions presented in this study are included in the article/Supplementary Materials. Further inquiries can be directed to the corresponding author.

## Supplementary Materials

The following supporting information can be downloaded at: https://www.mdpi.com/article/10.3390/ijms27104556/s1.

## Abbreviations

The following abbreviations are used in this manuscript:

AD	Alzheimer’s disease
LDL	low density lipoprotein
LMDs	lipid metabolism disorders
PPI	protein–protein interaction
EPC	Edge Percolated Component
MR	Mendelian randomization
MD	molecular dynamics simulation
RMSD	root-mean-square deviation
BBB	blood–brain barrier
OB	oral bio-availability
RO5	the Rule of Five
GWAS	genome-wide association studies
SNPs	single nucleotide polymorphisms
IVs	instrumental variables
LD	linkage disequilibrium
IVW	the inverse variance weighted
GO	Gene Ontology
BPs	biological processes
CCs	cellular components
MFs	molecular functions
KEGG	Kyoto Encyclopedia of Genes and Genomes
GPCRs	G protein-coupled receptors
MAFLD	Metabolic dysfunction-Associated Fatty Liver Disease
SMC	smooth muscle cell
VSMC	vascular smooth muscle cell
VEGF	vascular endothelial growth factor
MEK	Raf-mitogen-activated protein kinase kinase
ERK	extracellular signal-regulated kinase
HIF-1α	hypoxia-inducible factor-1α
GLUT1	glucose transporter 1
ROS	reactive oxygen species
CTD	component–target–disease
SAR	structure–activity relationship
S1P	sphingosine-1-phosphate
HDL	high-density lipoprotein
PI3K	phosphatidylinositol 3-kinase
HSPs	heat shock proteins
LDL-C	low density lipoprotein cholesterol
APP	amyloid precursor protein
AChE	acetylcholinesterase
SE	Standard Errors
