# Supplementary material for: Network Pharmacology Reveals the Therapeutic Potential of BBB-Permeable Compounds from Lonicera caerulea for Alzheimer’s Disease and Lipid Metabolism Disorders
Source: Int J Mol Sci. 2026 May 19;27(10):4556. doi: 10.3390/ijms27104556 (PMC13208057; doi:10.3390/ijms27104556)
Supplement: Supplementary file 1 [file ijms-27-04556-s001.zip › ijms-4234736-supplementary.pdf]

Supplementary material

A

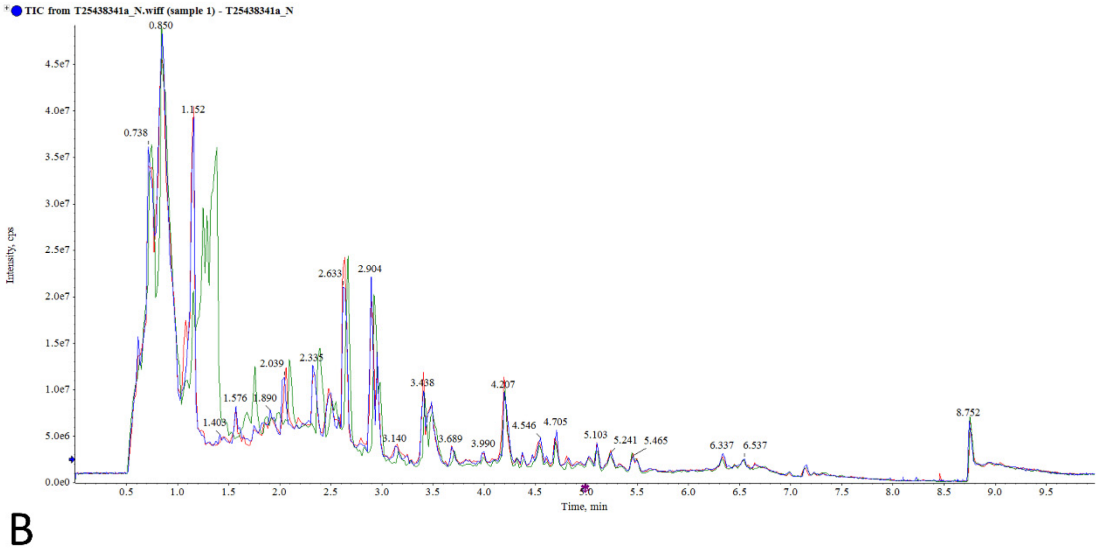

B

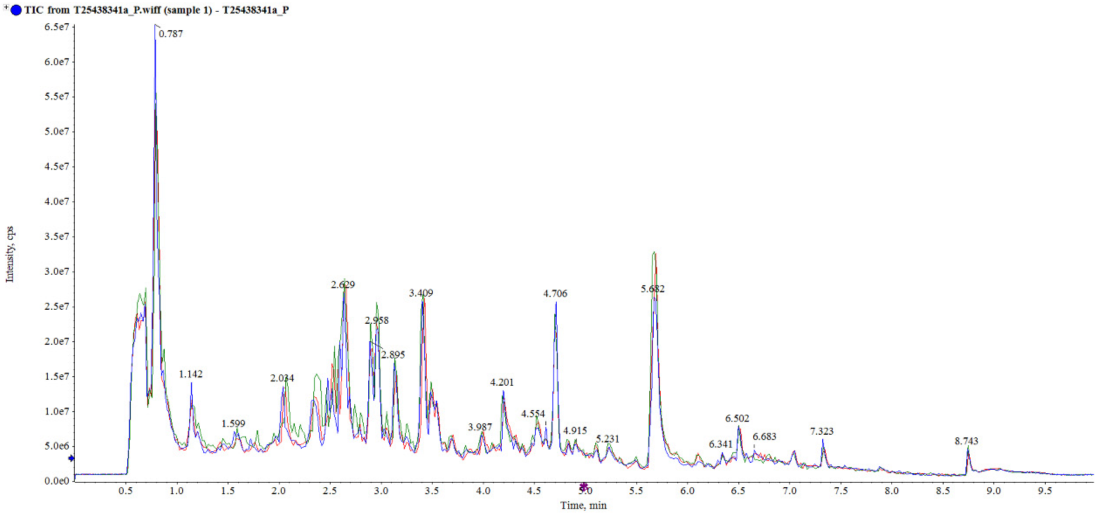

Figure S1 QC sample mass spectrometry detection TIC overlap map

As can be seen from Figure S1A-B, Lonicera caerulea is rich in a variety of active components as determined by HPLC-MS.

Table S1 The sources of 156 active ingredients in Lonicera caerulea.

No.	Compounds	DATA SOURCES
1	Cyanidin	literatures
2	Peonidin	literatures

No.	Compounds	DATA SOURCES
3	Delphinidin	literatures
4	Pelargonidin	literatures
5	Cyanidin-3-O-glucoside	literatures
6	Cyanidin-3-O-rutinoside	literatures
7	Cyanidin-3,5-O-diglucoside	literatures
8	Peonidin-3-O-glucoside	literatures
9	Cyanidin-3,5-O-dihexoside	literatures
10	Peonidin-3,5-O-dihexoside	literatures
11	Delphinidin-3-rutinoside	literatures
12	Delphinidin-3-p-coumaroylglucoside	literatures
13	Catechin	literatures
14	Isoquercetin	literatures
15	Apigenin	literatures
16	Diosmetin 3-O-glucoside	literatures
17	Kaempferol 3-O-sambubioside	literatures
18	Diosmetin-3-O-rutinoside	literatures
19	Quercetin 3-O-rutinoside	literatures
20	Apigenin 7-O-rutinoside	literatures
21	Kaempferol 3-O-rutinoside	literatures
22	Quercetin 3-O-rhamnoside	literatures
23	Kaempferol 7-O-rutinoside	literatures

No.	Compounds	DATA SOURCES
24	Isorhamnetin 7-O-rutinoside	literatures
25	Kaempferol 3-O-neohesperidoside	literatures
26	Neochlorogenic acid	literatures
27	Cryptochlorogenic acid	literatures
28	Dicaffeoylquinic acid	literatures
29	Quinic acid	literatures
30	Malic acid	literatures
31	Tartaric acid	literatures
32	Oxalic acid	literatures
33	Eucalyptol	literatures
34	Campesterol	literatures
35	α- & β-Amyrin	literatures
36	Verbenalin	literatures
37	Secologanic acid	literatures
38	Loganic Acid	literatures
39	7-Epi-Loganic Acid 7-O-Pentoside, 7-epi LAp	literatures
40	Sweroside	literatures
41	Rhamnoside	literatures
42	Phlorigidoside B	literatures
43	Pelargonidin 3-rutinoside	literatures
44	Trolox	literatures
45	Pelargonidin 3-glucoside ion	literatures
46	Cyanidin-3-glucoside	literatures
47	Quercetin-3-galactoside	literatures

No.	Compounds	DATA SOURCES
48	Pelargonidin-3-O-glucoside	literatures
49	cyanidin 3-O-beta-D-glucopyranoside	literatures
50	elagic acid	literatures
51	cyanidin-3-sophoroside	literatures
52	cyanidin-3-rutinoside	literatures
53	pelargonidin-3-rutinoside	literatures
54	Epicatechin	literatures
55	3-Caffeoylquinic acid	literatures
56	Fisetin	literatures
57	nobiletin	literatures
58	Dracylic acid	literatures
59	p-hydroxybenzoic acid	literatures
60	p-Coumaric acid	literatures
61	β-sitosterol	literatures
62	Myricetin3-O-galactoside	literatures
63	Luteolin-7-O-glucoside	literatures
64	Peonidin-3-glucoside	literatures
65	3,5-Dicaffeoylquinic acid	literatures
66	hydroxycinnamic acids	literatures
67	2,5-dihydroxybenzoic acid	literatures
68	Isoguanine	assays
69	Delphinidin-3-sambubioside	assays
70	Kaempferol	literatures&assays
71	Luteolin	literatures&assays

No.	Compounds	DATA SOURCES
72	Dihydroquercetin	literatures&assays
73	Quercetin	literatures&assays
74	Isorhamnetin	literatures&assays
75	Luteolin-7-glucoside	literatures&assays
76	Chlorogenic acid	literatures&assays
77	Caffeic acid	literatures&assays
78	Gallic acid	literatures&assays
79	Vanillic acid	literatures&assays
80	Citric acid	literatures&assays
81	Shikimic acid	literatures&assays
82	Loganin	literatures&assays
83	diosmin	literatures&assays
84	Rutin	literatures&assays
85	succinic acid	literatures&assays
86	Ferulic acid	literatures&assays
87	Hyperoside	literatures&assays
88	Baicalin	assays
89	Naringenin	assays
90	Protocatechuic acid	assays
91	4,5-di-Caffeoylquinic acid	assays
92	1-Caffeoylquinic acid	assays
93	Secologanin	assays
94	Palmatine	assays
95	7,11-Dehydromatrin	assays
96	Colchicine	assays

No.	Compounds	DATA SOURCES
97	Paeonolide	assays
98	Docosyl acetate	assays
99	Stypandrol	assays
100	Canaliculatol	assays
101	Rehmaionoside C	assays
102	kaempferol 7-O-glucoside	assays
103	Prim-O-glucosylcimifugin	assays
104	Kaempferol-7-rhamnoside	assays
105	Maltopentaose	assays
106	Brucine	assays
107	Gentiobiose	assays
108	Cellotetraose	assays
109	Forsythiaside	assays
110	Rhapontin	assays
111	Luteolin 7-glucuronide	assays
112	Procyanidin B1	assays
113	Procyanidin B2	assays
114	Epigallocatechin 3,3',-di-O-gallate	assays
115	Safflor yellow A	assays
116	Plantaginin	assays
117	10-Deacetylbaccatin III	assays
118	Irisxanthone	assays
119	Didymin	assays
120	Mangiferin	assays
121	Daphnoretin	assays

No.	Compounds	DATA SOURCES
122	Cellobiose	assays
123	Rishitin	assays
124	Neocarthamin	assays
125	Aminopterin	assays
126	Citicoline	assays
127	Zingerone	assays
128	Mulberrofuran A	assays
129	Oxoglutaric acid	assays
130	Adenine	assays
131	Biflorin	assays
132	Maltitol	assays
133	Sambunigrin	assays
134	Inosine	assays
135	Sennoside B	assays
136	Trehalose	assays
137	2-Pyrocatechuic acid	assays
138	Procyanidin B4	assays
139	Matairesinol	assays
140	Salicin	assays
141	Hesperidin	assays
142	Raffinose	assays
143	Neocarlinoside	assays
144	Globotriose	assays
145	D-Mannose	assays
146	Glucaric acid	assays

No.	Compounds	DATA SOURCES
147	Demethylwedelolactone	assays
148	Verbasoside	assays
149	Morin	assays
150	Laricitrin	assays
151	Methylparaben	assays
152	Nepitrin	assays
153	Azelaic acid	assays
154	Celastrol	assays
155	Podofilox	assays
156	Genistin	assays

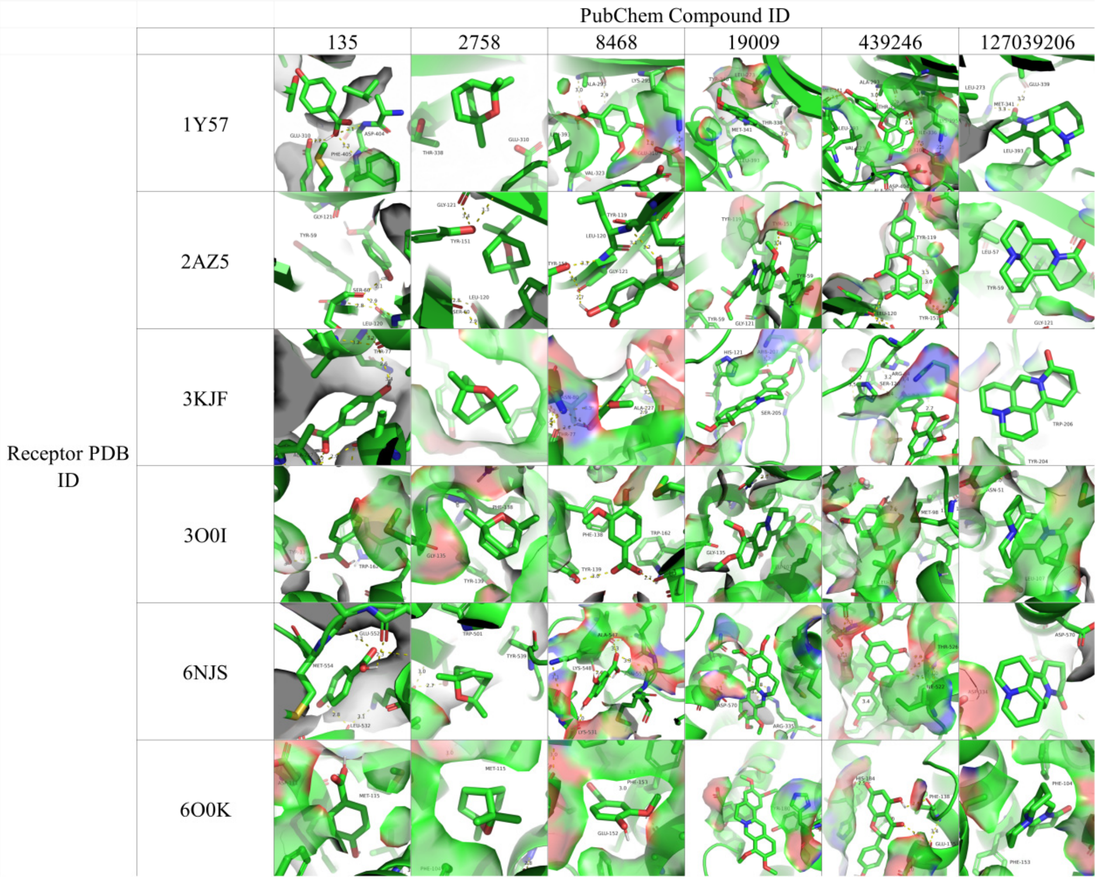

Figure S2 3D diagram of docking of six active compounds with six key receptors

Table S2 PubChem ID of each compound.

Compound	PDB ID
p-hydroxybenzoic	135
Eucalyptol	2758
Vanillic acid	8468
Palmatine	19009
Naringenin	439246
7,11-Dehydromatrin	127039206

Table S3 Center coordinates and size parameters of the docking grid box.

Protein	center_x	center_y	center_z	size_x	size_y	size_z
SRC	17.286	36.035	40.545	46	50	66
TNF	-20.210	77.847	35.334	70	58	58
CASP3	25.373	-8.433	9.179	46	36	70
HSP90AA1	-2.259	-12.681	-24.734	40	44	40
STAT3	0.790	38.847	0.138	70	58	86
BCL2	-18.548	2.259	-11.887	40	40	74

Table S4 Binding energy of docking of 6 proteins and 6 components respectively.

	SRC	TNF	CASP3	HSP90AA1	STAT3	BCL2
7,11-DEHYDROMATRIN	-7.6	-7.4	-6.2	-8.1	-6.8	-7.3
P-HYDROXYBENZOIC	-5.1	-5.1	-5.0	-6.6	-5.3	-5.3
PALMATINE	-7.8	-7.8	-6.8	-8.9	-7.0	-7.0
EUCALYPTOL	-5.5	-5.8	-4.3	-4.9	-4.7	-5.7
NARINGENIN	-8.1	-7.5	-6.9	-9.0	-7.3	-7.3
VANILLIC ACID	-5.3	-5.3	-5.2	-6.7	-5.3	-5.5

Table S5 The number of hydrogen bonds docked by 6 proteins and 6 components respectively.

	SRC	TNF	CASP3	HSP90AA1	STAT3	BCL2
7,11-DEHYDROMATRIN	0	0	0	0	0	1
P-	0	1	0	0	0	0

HYDROXYBENZOIC						
PALMATINE	0	0	0	1	0	0
EUCALYPTOL	0	0	0	0	0	0
NARINGENIN	1	1	0	0	2	0
VANILLIC ACID	1	0	0	0	1	0

For the three complex systems Naringenin-CASP3, Naringenin-STAT3, and 7,11-Dehydromatrin-BCL2, molecular dynamics simulations were performed for 100 nanoseconds each, with the results shown in Figure S3. The simulations demonstrated that in these systems, the RMSD value between the ligands and the protein backbone carbon atoms remained approximately 2.5 Å, with fluctuations controlled within 0.5 Å, and the shape of the curve was mainly similar to that of the protein backbone carbon atoms themselves, suggesting that the ligand fluctuations may be caused by the fluctuation of the protein. The RMSD value for the protein backbone carbon atoms was approximately 1.78 Å, with fluctuations of about 0.35 Å, indicating stable conformational states. Hydrogen bond analysis revealed that the three systems—Naringenin-CASP3, Naringenin-STAT3, and 7,11-Dehydromatrin-BCL2—formed one, two, and one stable hydrogen bond, respectively. No stable hydrogen bond donor-acceptor pairs were observed in the Naringenin-CASP3 and 7,11-Dehydromatrin-BCL2 systems, indicating that hydrogen bonds play only a secondary role in these binding modes.

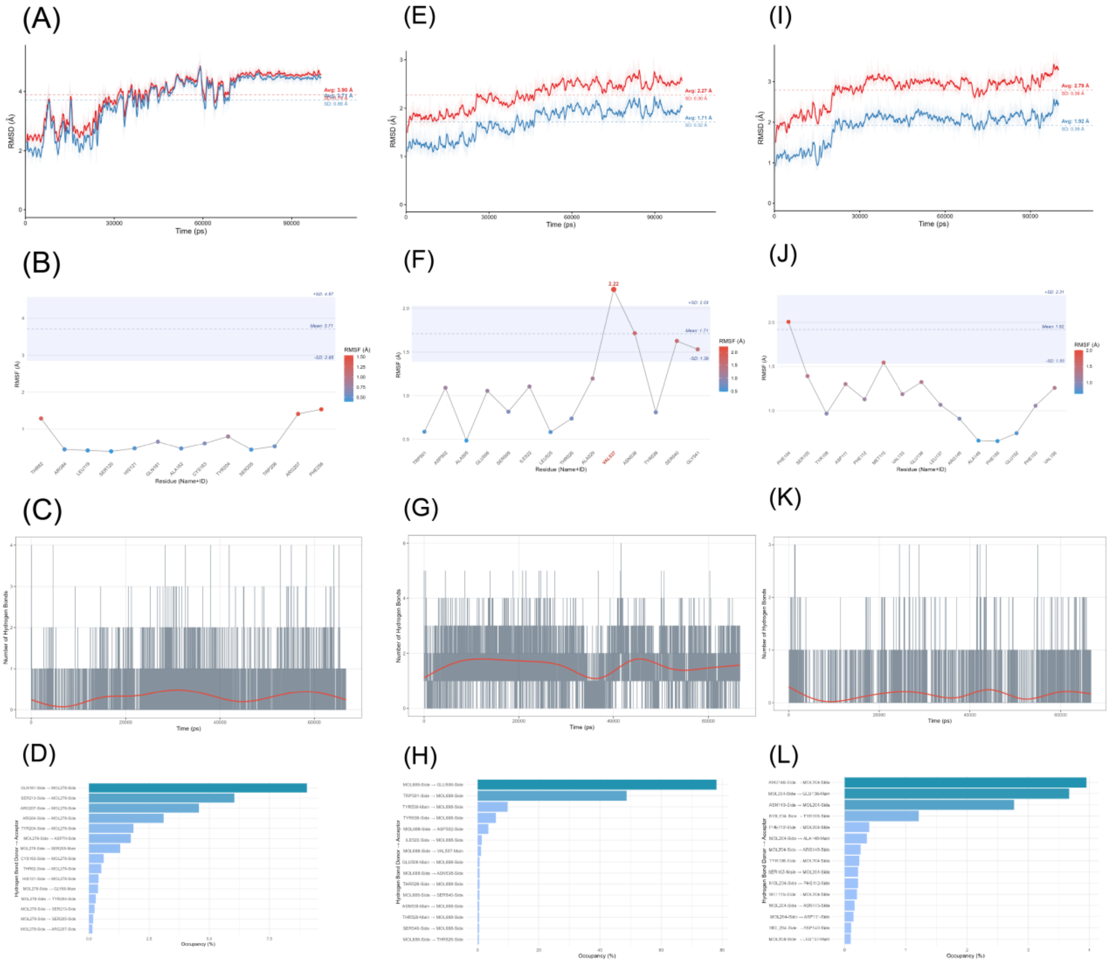

Figure S3. Analysis of the stability and interactions from molecular dynamics simulations of three complex systems. **(A)** Plot of the root-mean-square deviation (RMSD) over time for the Naringenin-CASP3 system, where the blue curve represents the intrinsic motion of the

protein backbone carbon atoms, and the red curve represents the motion of the ligand relative to the protein backbone carbon atoms. The thick lines represent the average of every 50 adjacent frames, which is used to suppress thermal noise; the thin lines in the background represent the raw data. **(B)** In Naringenin-CASP3 system, the root means square fluctuation (RMSF) distribution of each residue in the ligand binding pocket region, and the color of data points from blue to red indicates that the RMSF value is from low to high; The blue band at the top shows the mean and standard deviation of RMSD of protein skeleton carbon atoms. **(C)** The number of hydrogen bonds between protein and ligand in Naringenin-CASP3 system changes with simulation time, and the red curve is the statistical fitting result based on the generalized additive model (GAM). **(D)** The time occupation of hydrogen bonds between various residues and ligands in Naringenin-CASP3 system. **(E)** RMSD evolution curve over time for the Naringenin-STAT3 system. **(F)** Conformation overlay of the STAT3 protein during the simulation; the protein is depicted in cartoon form and colored according to simulation time (red-white-blue corresponds to the progression of the simulation from early to late stages). **(G)** Changes in the number of protein-ligand hydrogen bonds in the Naringenin-STAT3 system over simulation time. **(H)** The time distribution of hydrogen bond formation between residues and ligands in the Naringenin-STAT3 system. **(I)** RMSD evolution curves for the 7,11-Dehydromatrin-BCL2 system over time. **(J)** RMSF distribution of each residue in the ligand binding pocket region in 7,11-Dehydromatrin-BCL2 system. **(K)** The number of hydrogen bonds between protein and ligand in 7,11-Dehydromatrin-BCL2 system changed with simulation time. **(L)** The time occupation of hydrogen bond between each residue and ligand in 7,11-Dehydromatrin-BCL2 system.
